# Prophylaxis in healthcare workers during a pandemic: a model for a multi-centre international randomised controlled trial using Bayesian analyses

**DOI:** 10.1186/s13063-022-06402-w

**Published:** 2022-06-27

**Authors:** Pepa Bruce Metadata, Kate Ainscough, Lee Hatter, Irene Braithwaite, Lindsay R. Berry, Mark Fitzgerald, Thomas Hills, Kathy Brickell, David Cosgrave, Alex Semprini, Susan Morpeth, Scott Berry, Peter Doran, Paul Young, Richard Beasley, Alistair Nichol

**Affiliations:** 1 Medical Research Institute of New Zealand, Private Bag 7902, Newtown, Wellington 6242 New Zealand; 2 University College Dublin - Clinical Research Centre at St. Vincent’s University Hospital, Dublin, Ireland; 3 Berry Consultants LLC, Austin, TX USA; 4 Auckland District Health Board, Auckland, New Zealand; 5 National University of Ireland, Galway, Ireland; 6 University Hospital Galway, Galway, Ireland; 7 Counties Manukau District Health Board, Auckland, New Zealand; 8 Monash University - Australian and New Zealand Intensive Care Research Centre, Melbourne, Australia; 9 Department of Intensive Care, Alfred Hospital, Melbourne, Australia

**Keywords:** Prophylaxis, Healthcare worker, COVID-19, Bayesian analysis

## Abstract

**Background:**

Coronavirus disease 2019 (COVID-19) has exposed the disproportionate effects of pandemics on frontline workers and the ethical imperative to provide effective prophylaxis. We present a model for a pragmatic randomised controlled trial (RCT) that utilises Bayesian methods to rapidly determine the efficacy or futility of a prophylactic agent.

**Methods:**

We initially planned to undertake a multicentre, phase III, parallel-group, open-label RCT, to determine if hydroxychloroquine (HCQ) taken once a week was effective in preventing severe acute respiratory syndrome coronavirus 2 (SARS-CoV-2) infection in healthcare workers (HCW) aged ≥ 18 years in New Zealand (NZ) and Ireland. Participants were to be randomised 2:1 to either HCQ (800 mg stat then 400 mg weekly) or no prophylaxis. The primary endpoint was time to Nucleic Acid Amplification Test-proven SARS-CoV-2 infection. Secondary outcome variables included mortality, hospitalisation, intensive care unit admissions and length of mechanical ventilation.

The trial had no fixed sample size or duration of intervention. Bayesian adaptive analyses were planned to occur fortnightly, commencing with a weakly informative prior for the no prophylaxis group hazard rate and a moderately informative prior on the intervention log hazard ratio centred on ‘no effect’. Stopping for expected success would be executed if the intervention had a greater than 0.975 posterior probability of reducing the risk of SARS-CoV-2 infection by more than 10%. Final success would be declared if, after completion of 8 weeks of follow-up (reflecting the long half-life of HCQ), the prophylaxis had at least a 0.95 posterior probability of reducing the risk of SARS-CoV-2 infection by more than 10%. Futility would be declared if HCQ was shown to have less than a 0.10 posterior probability of reducing acquisition of SARS-CoV-2 infection by more than 20%.

**Discussion:**

This study did not begin recruitment due to the marked reduction in COVID-19 cases in NZ and concerns regarding the efficacy and risks of HCQ treatment in COVID-19. Nonetheless, the model presented can be easily adapted for other potential prophylactic agents and pathogens, and pre-established collaborative models like this should be shared and incorporated into future pandemic preparedness planning.

**Trial registration:**

The decision not to proceed with the study was made before trial registration occurred.

## Background

On the 31st of December 2019, a cluster of pneumonia cases of unknown origin in Wuhan, China, was first reported to the World Health Organization (WHO) [1]. Subsequently, a novel pathogen, severe acute respiratory syndrome coronavirus 2 (SARS-CoV-2) was identified. Within 1 month, the WHO had declared coronavirus disease 2019 (COVID-19), the disease associated with SARS-CoV-2 infection, a public health emergency of international concern [2]. Within 6 months, more than 10 million cases and half a million deaths had been reported across 182 countries [3]. The rapid spread of COVID-19 dictated a rapid research response to assess and develop medications for treatment. Just as important, given the infectivity of COVID-19, was the need to identify effective prophylactic agents [4, 5].

This was particularly pertinent in the healthcare setting as, early in the pandemic, healthcare workers (HCW) accounted for between 11 and 32% of reported cases in countries around the world [6–10]. Subsequent studies have confirmed that frontline workers have a higher risk of SARS-CoV-2 infection, and up to sevenfold higher risk of developing severe disease [11–13]. Internationally, COVID-19 has completely overwhelmed many healthcare systems. It is clear that having an adequate number of HCWs to meet the demands imposed by COVID-19 on healthcare systems presents a major ongoing challenge. An infection in a HCW not only means that the person is not available to work, but also has a number of potential downstream effects. Firstly, HCWs are at risk of transmitting SARS-CoV-2 infection to vulnerable hospitalised patients in their care before they become symptomatic. Secondly, every HCW who is diagnosed with COVID-19 results in several other HCWs being unable to work due to self-isolation requirements or transmission between HCWs. Thirdly, infections in HCWs have negative consequences on staff morale impacting their ability to continue providing care. Most importantly, infection in a HCW puts that person at risk of a life-threatening illness, particularly in those who are older and have co-morbidities [14]. As a result, prevention of COVID-19 infections in HCWs was identified as a key strategic objective by the WHO [15].

It is critical to global pandemic research efforts that the potential efficacy of simple, oral, safe and low-cost prophylactic regimens for those at high risk is rapidly determined. At the time this study was designed, a potential candidate which met the criteria for medication repurposing towards treating or preventing COVID-19 was hydroxychloroquine (HCQ). HCQ alters the cellular receptor for SARS-CoV-2, can inhibit the entry of SARS-CoV-2 into the cell, and can inhibit both endocytosis and replication within cells [16–20]. Additionally, HCQ has previously been used effectively for malaria prophylaxis with a favourable side effect profile at low doses, concentrates in the lung tissues and has a pharmacokinetic profile that lends itself to weekly, rather than daily, dosing [21, 22]. It also has the benefit of being off-patent, cheap and widely available. These are all ideal attributes of a prophylactic agent.

Based on this evidence, we planned a randomised controlled trial to investigate the hypothesis that 400 mg of oral HCQ weekly (after an 800 mg HCQ loading dose) would be superior to no prophylaxis at reducing the risk of acquiring laboratory-confirmed SARS-CoV-2 infection in frontline HCWs caring for patients with known or suspected COVID-19 disease. The plan was to first initiate the study in New Zealand (NZ) and Ireland (IE) with the potential to expand to other countries. There were many unknown parameters with respect to developing the trial protocol and statistical analysis plan, including likely COVID-19 infection and hospitalisation rates in each country, infection rates of healthcare workers exposed to COVID-19, the efficacy of HCQ in preventing infection with COVID-19 and the identification of a clinically meaningful difference that would indicate efficacy of HCQ prophylaxis in healthcare workers. It became apparent that using a traditional frequentist statistical approach was unlikely to be successful in the uncertain and rapidly evolving setting of a pandemic. Instead, a Bayesian adaptive approach was planned as it allows a design to be established in the absence of information required for a traditional fixed trial, resulting in a robust trial providing interpretable results in a timely manner.

Ultimately, this study did not begin recruitment due to the marked reduction in COVID-19 cases in NZ and the emergence of international concerns regarding the efficacy and risks of the use of HCQ treatment in COVID-19 [23]. However, the rapid spread of COVID-19 highlights the need for pre-established protocol templates for pandemic trials, to prevent unnecessary delays to study initiation. The goal of this article is to present a model, which can be incorporated into future pandemic preparedness. It uses HCQ and COVID-19 as examples, as initially intended, but these can easily be substituted for other prophylactic agents as well as both novel pathogens and well-recognised viruses, such as influenza. Moreover, the model illustrates how Bayesian analysis might be used in randomised controlled trials (RCT) of comparative efficacy in an emergent pandemic when the usual parameters that would inform fixed designs are unknown, and rapid assessment of efficacy or futility is essential.

## Methods

### Objectives

The trial was designed to determine if weekly directly observed therapy (DOT) with oral HCQ was superior to no prophylaxis at reducing acquisition of SARS-CoV-2 infection among HCWs working in facilities caring for patients with known or suspected COVID-19 disease.

#### Primary aim

- To assess whether HCQ prophylaxis reduced the incidence of SARS-CoV-2 infection in at risk HCWs

#### Secondary aims

- To assess whether HCQ prophylaxis reduced the incidence of hospitalisation with SARS-CoV-2 infection, or hospitalisation for any cause in at risk HCWs
- To assess whether HCQ prophylaxis reduced the incidence of intensive care unit (ICU) admissions with SARS-CoV-2 infection, or ICU admissions for any cause in at risk HCWs
- To assess whether HCQ prophylaxis reduced the incidence of admission with SARS-CoV-2 infection where mechanical ventilation was administered, or admission where mechanical ventilation was administered for any reason in at risk HCWs
- To assess whether HCQ prophylaxis reduced mortality due to SARS-CoV-2 infection, or all-cause mortality in at risk HCWs
- To assess whether HCQ prophylaxis reduced the duration of hospitalisation, ICU admission or mechanical ventilation with SARS-CoV-2 in at risk HCWs

### Trial design

Initially intended as a bi-national trial with multi-national potential, this was designed as a phase III, randomised, parallel-group, open label, one-sided superiority clinical trial with reference to the SPIRIT checklist [24]. The plan was to randomise participants 2:1 to HCQ or no prophylaxis.

### Trial setting

Healthcare facilities (including hospitals, residential care homes and primary care centres) in NZ, IE and subsequently selected international sites, where patients with COVID-19 were being treated.

#### Site initiation

Once the participating healthcare facility had at least one patient with Nucleic Acid Amplification Test (NAAT)-confirmed SARS-CoV-2 infection, they would start screening potential participants. Based on trial simulations, each site was to commence recruitment once the community attack rate exceeded 0.027% per week.

### Eligibility criteria

#### Inclusion criteria

- Adults aged ≥ 18 years
- Working in healthcare facilities (including hospitals, residential care homes and primary care centres) that have had at least one patient with NAAT-confirmed SARS-CoV-2 infection. Specifically, this would include doctors, dentists, nurses, midwives, allied health, science and technical professions (e.g. pharmacists, therapists, technicians), non-regulated workers (e.g. carers, support workers) and non-clinical staff (e.g. cleaners, orderlies, administrative staff).
- Willing and able to give informed consent for participation in the trial.
- In the investigator’s opinion, able and willing to comply with trial requirements.

#### Exclusion criteria

Participant report of:

- Current or previous diagnosis of SARS-CoV-2 infection
- Current symptoms of SARS-CoV-2 infection
- Current use of HCQ or chloroquine
- Any known or suspected contra-indications or cautions to HCQ or chloroquine use (Table 1)
- Congenital or acquired QT prolongation, or known risk factors:
  - ◦ age >75
  - ◦ renal or hepatic disease
  - ◦ uncorrected hypokalaemia and/or hypomagnesaemia
  - ◦ cardiac disease, e.g., heart failure, ischaemic heart disease, cardiomyopathy
  - ◦ proarrhythmic conditions, e.g., bradycardia (< 50 bpm)
  - ◦ a history of ventricular dysrhythmias (ventricular tachycardia or ventricular fibrillation)
  - ◦ family history of QT prolongation or sudden cardiac death
  - ◦ concomitant use of QT prolonging agents (Table 2)
- In females of child bearing age;
  - ◦ current pregnancy
  - ◦ breastfeeding
  - ◦ planned pregnancy during the course of the trial
  - ◦ not taking measures to avoid pregnancy
- Any other condition which, at the Investigator’s discretion, may present a safety risk or impact the feasibility of the study or the study results.

**Table 1:** Contra-indications and cautions to HCQ or chloroquine use

**Current diagnosis of:**			
Maculopathy of the eye			
Hypersensitivity to 4-aminoquinoline compounds			
Porphyria			
Psoriasis			
Severe gastrointestinal disorders (such as inflammatory bowel disease)			
Severe neurological disorders			
Severe blood disorders			
Epilepsy			
Diabetes mellitus on pharmacological treatment			
Sensitivity to quinine			
G6PD deficiency			
**Concurrent use of:**			
Agalsidase	Halofantrine	Phenobarbital	Refapentine
Carbamazepine	Monoamine oxidase inhibitors	Phenytoin	Rifampicin
Cisapride	Metamizole	Praziquantel	St John’s wort
Cyclosporin	Mexiletine	Primidone	Tamoxifen
Digoxin	Neostigmine	Pyridostigmine

**Table 2:** Concurrent medications identified as having risk of QT prolongation and/or Torsades de Pointes

Amiodarone	Dofetilide	Levofloxacin	Quetiapine
Amisulpride	Dolasetron	Levomepromazine	Quinidine
Amitriptyline	Domperidone	Lithium	Ranolazine
Atazanavir	Erythromycin	Lopinavir/ritonavir	Risperidone
Azithromycin	Escitalopram	Maprotiline	Sevoflurane
Bedaquiline	Flecainide	Methadone	Sulpiride
Bendroflumethiazide	Fluconazole	Metoclopramide	Tacrolimus
Bepridil	Fluoxetine	Mianserin	Telithromycin
Betrixaban	Fluvoxamine	Mirtazapine	Thioridazin
Buprenorphine	Furosemide	Moxifloxacin	Tiapride
Chlorpromazine	Granisetron	Nicardipine	Tizanidine
Cimetidine	Haloperidol	Nortriptyline	Torasemide
Ciprofloxacin	Hydrochlorothiazide	Ofloxacin	Tramadol
Citalopram	Hydrocodone	Ondansetron	Trazodone
Clarithromycin	Hydroxyzine	Paloperidone	Trimipramine
Clofazimine	Iloperidone	Paroxetine	Venlafaxine
Clomipramine	Imipramine	Perphenazine	Voriconazole
Clozapine	Indapamide	Pimozide	Ziprasidone
Delamanid	Itraconazole	Posaconazole	Zotepine
Desipramine	Ivabradine	Prochlorperazine	Zuclopenthixol
Dexmedetomidine	Ketoconazole	Propafenone	
Disopyramide	Lacidipine	Propofol	

### Interventions

#### Choice of comparators

This study was to compare HCQ taken orally, at doses traditionally used for malaria suppression (a loading dose of 800 mg, followed by 400 mg HCQ weekly) vs. no prophylaxis [21]. Participants would have been required to attend a study clinic once a week on the same day, ± 2 days if necessary. Due to the importance of expediency, it was not practical to manufacture placebo capsules or tablets of identical appearance and, therefore, no placebo prophylaxis was to be administered for those participants not randomised to the intervention.

#### Criteria for discontinuing allocated interventions

Each participant would have had the right to withdraw from the trial at any time, and the reason for withdrawal, if given, was to be documented. In addition, the investigators could have discontinued a participant from the allocated intervention at any time if they considered it necessary, for any reason, including:

- Development of an adverse event (AE) that required discontinuation of study intervention
- Development of a contraindication to study intervention
- Primary endpoint met (NAAT-proven SARS-CoV-2 infection)

#### Strategies to improve adherence

It was anticipated that HCWs would be highly motivated to take the study intervention. Medication would be dispensed weekly by DOT, and the importance of attendance at weekly clinic visits reinforced when dispensing occurred. This would provide the opportunity for all participants randomised to HCQ to be 100% compliant with their therapy and would prevent participants affecting the intervention through storing the medication, or sharing medication amongst their co-workers or family members.

In the event that a participant was unable to attend a study clinic in the allocated ± 2-day window (due to shift patterns, self-isolation etc.), a home pack consisting of 2 weeks supply of intervention would be utilised and DOT completed virtually.

### Concomitant care permitted

No restrictions were to be placed on concomitant care.

### Outcomes

#### Primary outcome variable

- Time to NAAT-proven diagnosis of SARS-CoV-2 infection. In this case, time is used as a surrogate for exposure to increased risk of contracting SARS-CoV-2 infection. Participants without SARS-CoV-2 infection at trial completion would be censored at that time.

#### Secondary outcome variables

- Mortality: survival time from randomisation until trial completion with participants lost to follow-up censored from the last documented contact time.
- Hospitalisation and ICU admissions: length of stay was to be calculated from randomisation until discharge, death or trial completion.
- Mechanical ventilation: length of mechanical ventilation was to be calculated from randomisation until removal from mechanical ventilation and / or death, or trial completion.

### Participant timeline

**Fig. 1:**
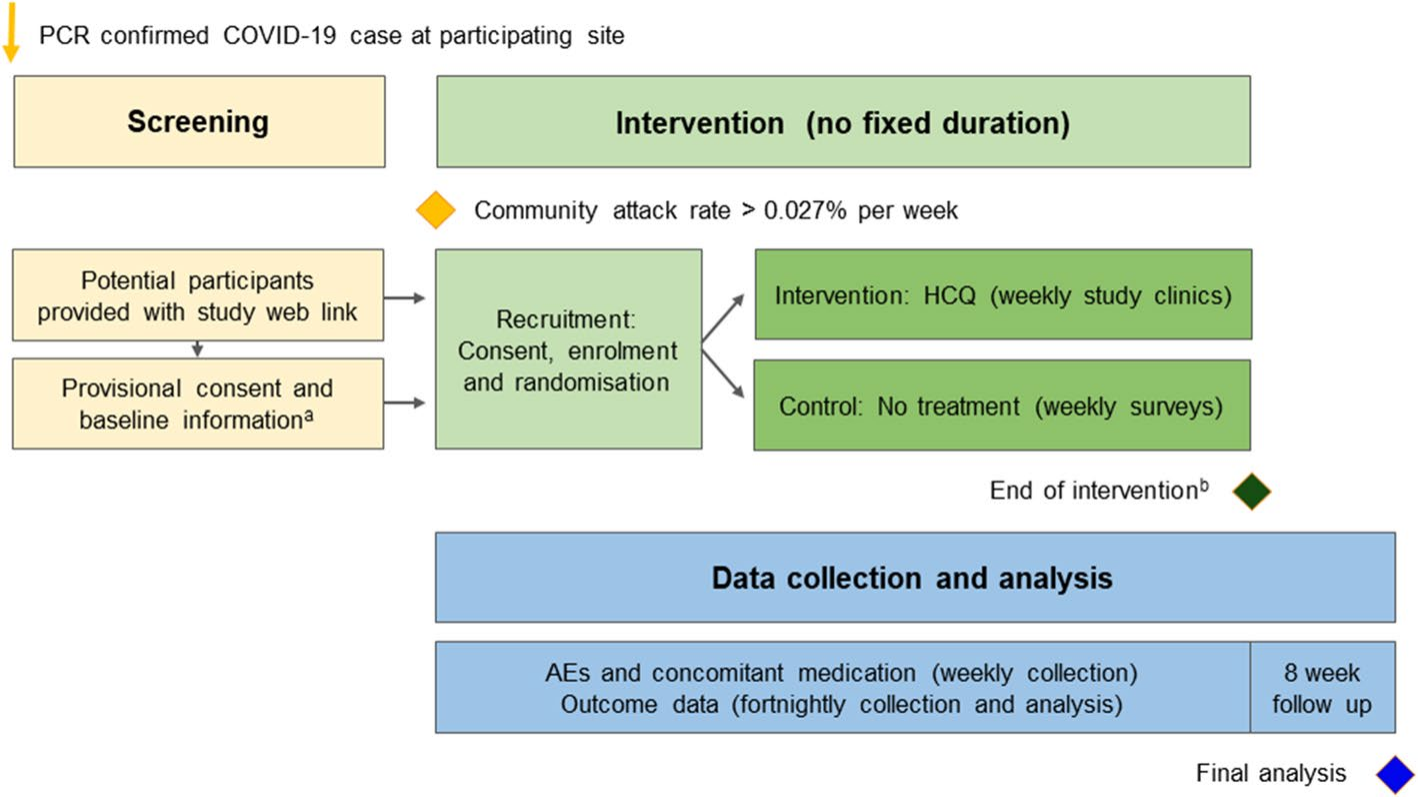
^a^NZ only - submitted directly via study web portal; ^b^End of intervention triggered if (i) adaptive analysis demonstrates efficacy or futility, (ii) HCQ supply is exhausted or, (iii) COVID-19 pandemic is controlled; PCR, Polymerase Chain Reaction; HCQ, hydroxychloroquine; AE, Adverse Event

The study was designed as a ‘trial in perpetuity’ (Fig. 1). Participants were to be initially enrolled for up to 12 months, with a review at 6 months, and repeat consent to be completed if extensions required. Study interventions were to be discontinued when one of the following occurred:

- Adaptive analysis demonstrated success or futility
- The supply of up to 1 million doses of HCQ was exhausted
- The pandemic was controlled in NZ, IE and all other participating countries, with no new cases occurring for 28 consecutive days (the trial would stop on this basis in an individual country but continue in other countries).

Safety and outcome data would continue to be collected on all participants for 8 weeks after they discontinued study interventions. The end of trial would therefore be 8 weeks after the final dose of HCQ was administered (as determined by the aforementioned prespecified rules). This was designed to reflect the long half-life of the study intervention and to capture all affected outcomes.

### Sample size

The trial had no fixed sample size and no fixed duration of intervention due to the limited data on which to base the trial design calculations and due to the ethical imperative to ensure as many HCWs as possible had access to the study intervention if they wished to receive it. Instead of using a fixed sample size, we planned to administer a maximum of 1 million doses of HCQ prophylaxis to frontline HCWs. The number of NAAT-proven diagnoses of SARS-CoV-2 infection throughout the trial would drive the total number of HCWs recruited and the duration of follow up.

### Trial procedures

#### *Recruitment (day* < *1)*

Recruitment was to be overseen by ICU research staff or other research departments within participating healthcare facilities. Invitations to participate were to be advertised through facility-wide newsletters and departmental meetings with potential participants provided with a web link to a study web portal, designed by Spiral Singular Software (Wellington, NZ), that provided study information and would allow participants to determine whether they were eligible for study participation.

Due to regional differences in data protection guidance and data management, plans to maximise recruitment varied between NZ and IE. In NZ, potential participants would submit their own baseline information and contact details into the study web portal. Eligible candidates that agreed to enrol in the study would then be able to provide provisional consent directly via the portal. The details provided would then be used to contact potential participants and arrange for enrolment to occur once the study was initiated in a particular healthcare facility. In IE, the web portal would not collect any baseline details. The portal would advise eligible candidates to contact their local research team to arrange an appointment in a designated safe area at their local facility.

#### Informed consent, enrolment and randomisation (day 1)

Potential participants would attend a study clinic where research staff would discuss the Participant Information Sheet-Consent Form and verify eligibility. If the participant agreed to enrol in the study, they would then provide written informed consent, including consent for study staff to contact their healthcare providers where necessary to obtain study outcome data, and the contact details of their General Practitioner (GP) would be documented. In NZ, participants’ national health index number would also be recorded. Participants would then be randomised and a letter would be sent to their GP to inform them of study enrolment and assigned intervention. For participants randomised to HCQ therapy, the 800 mg loading dose would be administered under DOT at the first clinic appointment.

#### Subsequent study contact (day > 1)

All participants randomised to the HCQ regimen were to attend study clinics for DOT once a week on the same day, ± 2 days, at a time and day convenient to both investigator and participant, for the duration of the study. At each clinic appointment, participants were to be asked to report any AEs through a standardised survey, and confirm they had not started any new medication that would make them ineligible to continue in the study (Tables 1 and 2). All participants randomised to no prophylaxis were to be contacted remotely, at weekly intervals, to report any AEs through the standardised survey.

### Assignment of interventions

#### Sequence generation

The allocation sequence was to be randomly determined by a computer algorithm. Randomisation was to be stratified by site with block randomisation and variable block sizes.

#### Concealment mechanism and implementation

Central randomisation was to be performed by the investigators on site, using a secure, web-based, randomisation interface designed by Spiral Singular Software. Randomisation was not to be performed until participants fulfilled all eligibility criteria, provided full written informed consent and were ready to be assigned to study interventions.

### Data collection and management

#### Plans for assessment and collection of outcomes

A summary schedule of data to be collected is shown in Table 3. Baseline data (Table 4), AE data and new concomitant medications were to be provided by participants and confirmed by the research team. DOT was to be recorded by the research team at each study clinic. Where possible, study outcome data were to be obtained from national databases (i.e. the NZ Ministry of Health as a direct data import) or regional databases (i.e. Hospital Human Resource Departments), with provisions to contact participants’ healthcare providers when required, and uploaded by the research team on a fortnightly basis to allow for planned adaptive analyses to occur. These processes were designed to adapt to privacy laws and institutional review boards at each participating site.

**Table 3:** Schedule of procedures

**Week**	**Screening**^a^	**Recruitment**^b^	**Weekly DOT Clinic Visits**		
	** ≤ 1**	**1**	**2**	**3**	***N***
Potential participants provided with study web-link	X				
Provisional informed consent submitted^c^	X				
Baseline information collected	X^c^	X			
Written informed consent		X			
Inclusion/Exclusion criteria verification		X			
Medical history and demographics verification		X			
National Health Index documented^c^		X			
GP Contact details documented		X			
Randomisation		X			
Review AEs			X	X	X
Confirm no ineligible concurrent medication			X	X	X
DOT administered^d^		X^e^	X	X	X
Inform GP of study enrolment		X			
In case of withdrawn, document cause and inform GP			X	X	
Inform GP of study completion					X

^a^Commenced once participating site had at least one patient with Nucleic Acid Amplification Test (NAAT)-confirmed SARS-CoV-2 infection

^b^Commenced once community attack rate exceeded 0.027% per week

^c^NZ only

^d^Intervention group only

^e^First DOT—loading dose of 800 mg of HCQ

*DOT* Directly observed therapy, *N* total duration of trial intervention in weeks

**Table 4:** Baseline information to be collected

Age
Gender
Ethnicity (NZ only): NZ European; Māori; Pacific Peoples; Other
Occupation: allied health, science and technical professional; dentist; doctor; midwife; non-clinical staff; non-regulated worker; nurse
Height and weight (for body mass index calculation)
Medical conditions: asthma, chronic cardiac disease (not hypertension), chronic neurological disorder, chronic pulmonary disease (not asthma), diabetes mellitus (diet-controlled), hypertension, malignancy, smoking status (current, ex-smoker, non-smoker)
As a measure of baseline risk, all potential participants were to be asked if they had been directly involved in the care of a patient with COVID-19 (i.e. direct contact with a patient with COVID-19 or their immediate surroundings)

#### Data management and confidentiality

As much data as possible was to be captured by e-source, through direct data capture into a Clinical Data Management Application (CDMA) developed by Spiral Singular Software. This trial was designed to allow the recruitment of many thousands of participants in a short space of time. Data collection methods take this, and the requirement that the shared study database conformed to individual data security and protection standards in all participating countries, into account.

The study database was to be formed of de-identified (pseudonymised) data. Each participant was to be allocated a unique participant code, which enabled the local research team at each site to keep a master list of recruited participants. These codes would be the local key to link participant data to their source data. The master list of codes was to be stored securely and would never leave the participating site. All source data were to be entered into the secure CDMA and only applicable de-identified study data transferred into a centralised secure electronic case report form.

### Statistical methods

#### Statistical model

The planned analysis of HCQ versus no prophylaxis was a Bayesian model of the time to NAAT-proven diagnosis of SARS-CoV-2 infection. The endpoint for subject $i$ on prophylaxis arm $d$ is represented as ${T}_{i,d}.$ For each subject and intervention group, the primary outcome is modelled as follows:

$${T}_{i,d}\sim Exp({\lambda }_{d})$$

where $d$ =0 indicates the no prophylaxis arm and $d$ =1 indicates the HCQ arm. The parameters ${\lambda }_{0}$ and ${\lambda }_{1}$ are the hazard rates for the no prophylaxis and HCQ arms, respectively. In this model, we specify ${\lambda }_{1}={\lambda }_{0}{e}^{\theta }$ where $\theta$ is the log hazard ratio for HCQ relative to no prophylaxis. The hazard ratio (HR) of HCQ versus no prophylaxis is defined as $\mathrm{exp}(\theta ).$

##### Prior distributions

The hazard rate for the no prophylaxis group has a weakly informative prior with a mean of 0.005 events per week; ${\lambda }_{0} \sim Gamma(1.0, 0.005).$ The log hazard ratio, $\theta ,$ has a moderately informative prior with a mean of ‘no effect’; $\theta \sim Normal(0.0, 0.52)$. This moderately informative prior centred on zero (no effect of HCQ) was selected based on results of clinical trial simulations to restrict the type I error rate of the proposed design.

##### Consideration of efficacy (Fig. 2)

**Fig. 2:**
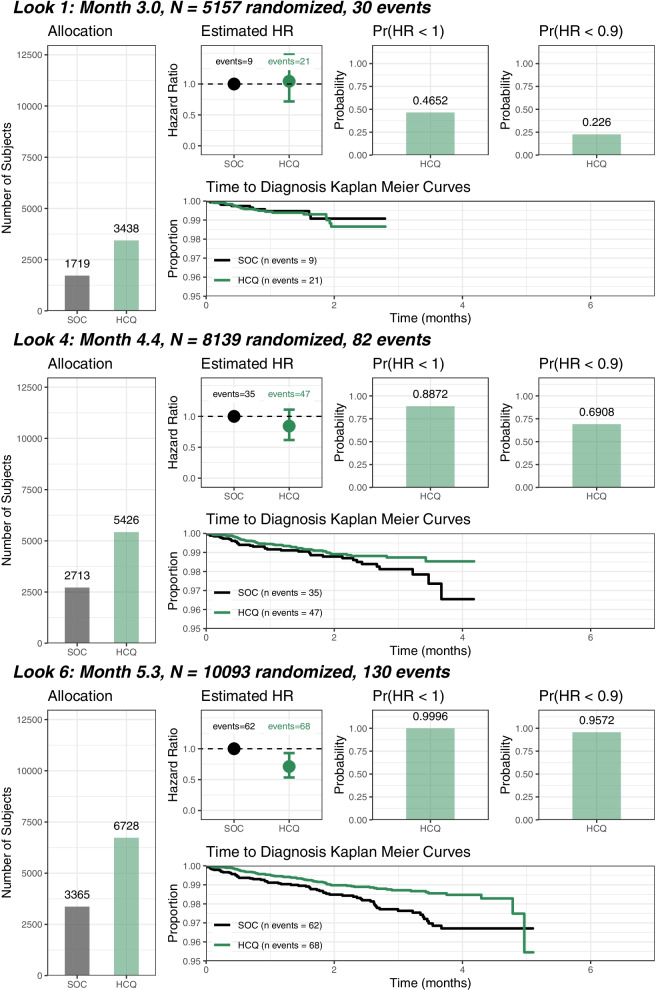
N, number of subjects; SOC, standard-of-care (no treatment); HCQ, hydroxychloroquine; Pr, Probability; HR, Hazard Ratio

Stopping for expected success: HCQ would have been declared superior to no prophylaxis if there was a greater than 0.975 posterior probability of reducing the risk of acquiring SARS-CoV-2 infection by more than 10% (i.e. HR < 0.9). If the trial was stopped for expected success, 8 weeks of follow-up would be completed to reflect the long half-life of HCQ.

Final success: HCQ would have been declared superior if after completion of follow-up, the prophylaxis had at least a 0.95 posterior probability of reducing the risk of acquiring SARS-CoV-2 infection by more than 10% (i.e. HR < 0.9).

##### Consideration of futility (Fig. 3)

**Fig. 3:**
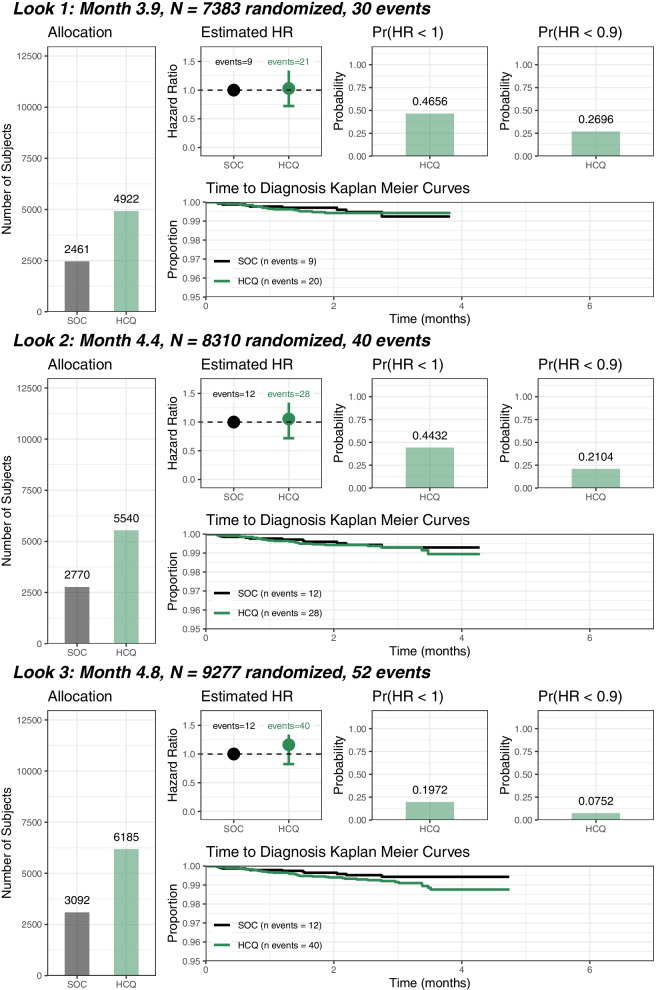
N, number of subjects; SOC, standard-of-care (no treatment); HCQ, hydroxychloroquine; Pr, Probability; HR, Hazard Ratio

Futility would have been declared if HCQ was shown to have less than a 0.10 posterior probability of reducing acquisition of SARS-CoV-2 infection by more than 20% (i.e. HR < 0.8).

##### Trial simulations and operating characteristics

**Fig. 4:**
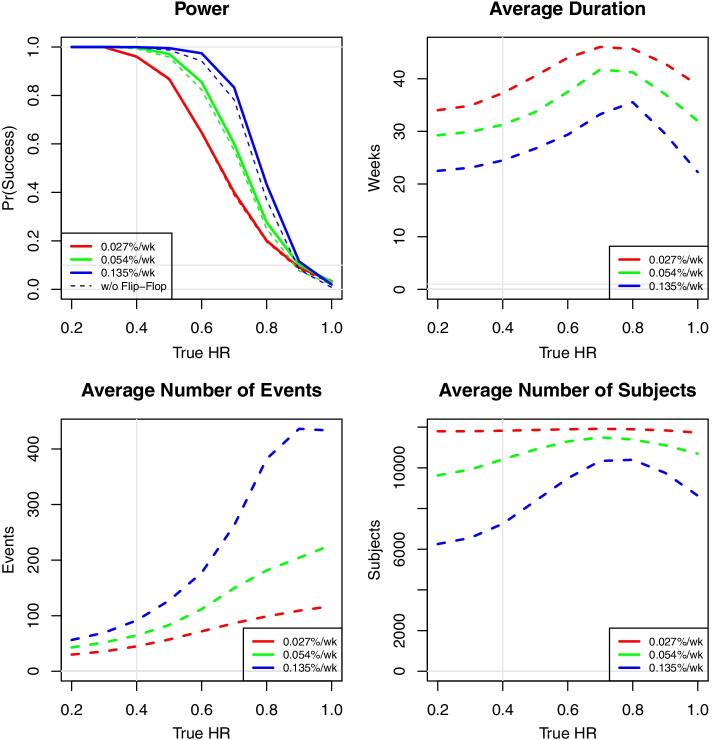
Operating characteristics of the trial design

The operating characteristics of the trial design are shown in Fig. 4. The proposed design was evaluated through simulation of thousands of trials under a range of possible scenarios for the accrual rate of HCWs, hazard rate for the no prophylaxis group and the HR effect of HCQ, in order to assess the operating characteristics of the statistical design.

The design was intended to have no fixed sample size, but for the purposes of simulation, a maximum sample size is required. For simulation purposes, a maximum of 12,000 HCW could be accrued, with a maximum follow-up time of 6 months following the last enrolled HCW. If this maximum time were to be reached, a final analysis was performed. Accrual was assumed to be relatively fast, reaching full accrual in approximately 6 months. The timing of adaptive analyses could be re-assessed if actual accrual was much slower than anticipated. Patient event times were then simulated from an exponential distribution depending on their intervention assignment and the scenario being simulated.

Eight scenarios were simulated for the SARS-Cov-2 infection event rate for the no prophylaxis group, given in units of the mean percentage of the HCW population that would be infected each week:

**Table:** 

0.0135%	0.0270%	0.0405%	0.0540%	0.0811%	0.108%	0.135%	0.162%

Nine scenarios were simulated for the HR for the HCQ prophylaxis group:

**Table:** 

0.2	0.3	0.4	0.5	0.6	0.7	0.8	0.9	1.0

All 72 pairs of scenarios from the control rate and HR were combined to provide a full range of scenarios.

**Fig. 5:**
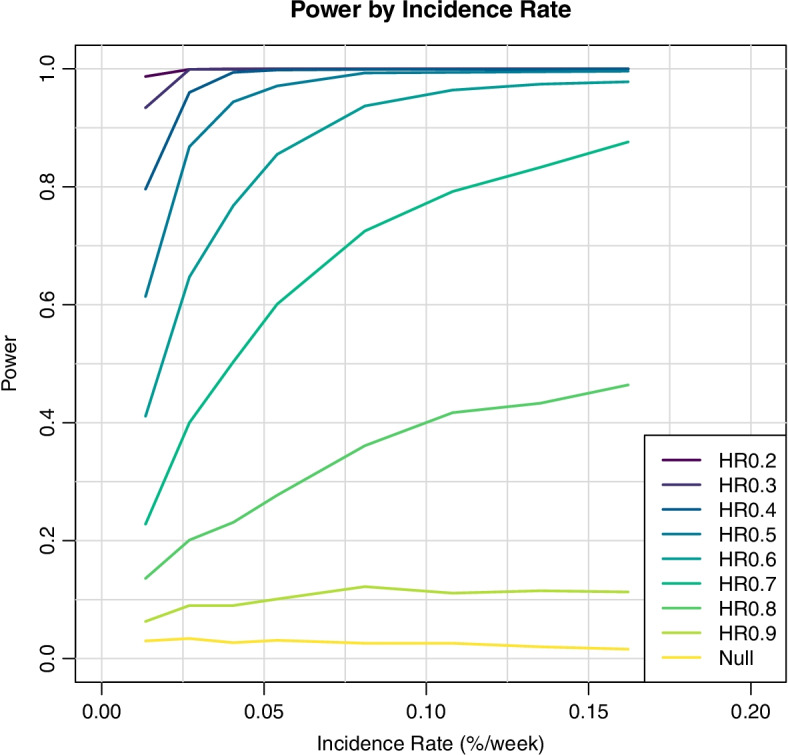
Statistical model power, by incidence rate. The simulated type I error rate when hydroxychloroquine (HCQ) has no prophylactic effect (hazard ratio = 1) ranges from less than 1 to 3% for a wide range of attack rates in the no prophylaxis group. If HCQ has a 10% reduction in the risk of acquisition of SARS-CoV-2 infection, the proposed design declares superiority of HCQ in less than 9% of simulated trials across scenarios

The statistical power of the design is summarized by control rate and HR in Fig. 5. The simulated type I error rate when HCQ has no prophylactic effect (HR = 1) ranges from less than 1 to 3% for a wide range of attack rates in the no prophylaxis group. If HCQ has a 10% reduction in the risk of acquisition of SARS-CoV-2 infection, the proposed design declares superiority of HCQ in less than 9% of simulated trials across scenarios. The attack rate plays a critical role in the power of the trial. If the attack rate was low (0.0135%), the trial would have been 80% powered to demonstrate superiority with a 0.40 HR for HCQ. If the event rate was high (0.162%), the trial would have been powered at 80% to detect a HR of 0.70 or better. We selected 0.027% SARS-CoV-2 infection event rate in HCWs on no prophylaxis as the threshold for site initiation because the design has > 80% power for the target HR of 0.5.

### Why a ‘super-superiority’ model?

The 97.5% probability of an effect of at least 10%, a condition that is more difficult than tradition efficacy, was chosen as a measure to restrict type I error and to increase the chance that successful trials were identifying clinically relevant effects. The rule also ties more closely with the futility rule, which is stopping trials when there is strong evidence of a minimal effect (20% or less).

### Statistical analysis

The statistical analysis was planned to use the intention-to-treat principle and to include all participants as they were randomised regardless of the intervention received or if the outcome was missing.

#### Timing of efficacy analyses

The first analysis was to occur after 15 diagnosis events had been recorded, and then every 2 weeks thereafter. At each adaptive analysis, the primary Bayesian model was to be fit to the accumulated data and the decision rules for early futility and success were to be assessed based on the resulting posterior probabilities [25–28]. The decision rules used for early success have been calibrated to restrict type I error [29].

#### Primary outcome

The Bayesian model is fitted to the data at each adaptive analysis, and the posterior distribution is estimated using Markov Chain Monte Carlo. The posterior distribution of the HR of HCQ to no prophylaxis was to be used to assess the a priori rules for success and futility at each adaptive analysis.

At the final analysis, a covariate-adjusted sensitivity analysis was to assess the robustness of the results from the primary analysis model. The Bayesian primary analysis model was to be extended to incorporate the following predictors into the log hazard rate: age, sex, ethnicity, BMI, smoking status (current, ex-smoker, non-smoker), baseline work risk for COVID-19 and the presence or absence of comorbidities including chronic cardiac disease (not hypertension), hypertension, diabetes mellitus (diet-controlled), chronic pulmonary disease (not asthma), asthma, chronic kidney disease, chronic neurological disorder and malignancy. The covariate adjustment takes the form of a Cox regression model, where the control infection rate is modelled as:

$${\lambda }_{0,i}={\lambda }_{0}{e}^{\sum \beta {X}_{i}}$$

for patient $i$ with covariates ${X}_{i}$.

#### Secondary outcomes

Mortality: survival time from randomisation until trial completion was to be assessed by Cox Proportional Regression, unadjusted initially, and then adjusted for age, sex, ethnicity, BMI, smoking status (current, ex-smoker, non-smoker), baseline work risk for COVID-19 and the presence or absence of comorbidities including chronic cardiac disease (not hypertension), hypertension, diabetes mellitus (diet-controlled), chronic pulmonary disease (not asthma), asthma, chronic kidney disease, chronic neurological disorder and malignancy. The assumption of proportional hazards was to be diagnosed via the Grambsch and Therneau proportionality test [30].

Hospitalisation and ICU admissions: length of stay was to be compared between the HCQ and the no prophylaxis group. Follow-up was to be limited to 28 days, with outcomes censored at 28 days. Deaths were to be assigned an outcome of censored at 30 days to represent an outcome worse than any survival. The length of stay was to be analysed as a time-to-event outcome, with a piecewise exponential hazard rate, allowing for differential rates by week of stay.

Mechanical ventilation: length of mechanical ventilation was to be compared between the HCQ and the no prophylaxis group. This outcome was to be modelled in similar fashion to hospitalisation time.

### Oversight and monitoring

#### Data monitoring committee

An independent Data and Safety Monitoring Committee (DSMC) was established to oversee the conduct of the trial and verify the correct implementation of the clinical trial design by reviewing AEs and early results at adaptive analyses as well as enrolments and withdrawals, to ensure adequate study safety, and minimal risk to participants. Where necessary, the DSMC was to make recommendations which could include early termination, suspension or modification of the trial, or changes in consent processes. The DSMC for this trial included internationally recognised experts in internal medicine, clinical trials and Bayesian trial design.

#### Adverse event reporting

Use of HCQ at a dose of 400 mg per week is well established for malaria prophylaxis and has a favourable side effect profile supported by decades of use. The risk of major side effects when used in this was is very low.

For the purposes of this study, data was to be collected relating to (i) adverse reactions to study medication, (ii) hospitalisation due to events associated with exposure to HCQ prophylaxis and (iii) death. A subset of pre-defined AEs, including classification of serious adverse events as detailed in Table 5, would be reviewed in the weekly standardised survey. Any pregnancy occurring during the clinical trial and the outcome of the pregnancy would be recorded and followed up for congenital abnormality or birth defect, at which point it would fall within the definition of ‘serious’.

**Table 5:** Pre-defined adverse events

**Symptoms of:**	
Eye	Blurring of vision
Ear and labyrinth	Hearing loss, tinnitus, vertigo
Gastrointestinal	Abdominal pain, diarrhoea, nausea, vomiting
Immune system	Angioedema, bronchospasm, urticaria
Metabolism	Anorexia, lethargy, weight loss
Musculoskeletal	Muscle weakness
Nervous system	Ataxia, convulsions, dizziness, headache
Psychiatric	Affect lability, nervousness, nightmares, suicidal thoughts
Skin	Alopecia, bleaching of hair, pigmentary changes, pruritus, skin rashes
**New diagnosis of:**	
Blood/Lymphatic	Agranulocytosis, anaemia, bone marrow depression, leucopoenia, thrombocytopenia
Cardiac	Cardiomyopathy, QT interval prolongation
Eye	Corneal changes, extraocular muscle palsies, maculopathies, retinopathy
Hepatobiliary	Abnormal LFTs, fulminant hepatitis
Metabolism	Hypoglycaemia
Musculoskeletal/connective tissue	Absent or hypoactive deep tendon reflexes, neuromyopathy, sensorimotor disorders
Nervous system	Extrapyramidal disorders, nerve deafness, nystagmus
Psychiatric	Psychosis,
Skin	Porphyria, psoriasis
**Serious adverse events:**	
	Results in death
	Is life-threatening
	Requires inpatient hospitalisation or prolongation of existing hospitalisation
	Results in persistent or significant disability/incapacity
	Consists of a congenital anomaly or birth defect
	Other ‘important medical events’ may also be considered serious if they jeopardise the participant or require an intervention to prevent one of the above consequences

## Discussion

This clinical trial was designed in response to the ethical imperative to identify a simple, oral, safe and low-cost prophylactic regimen for HCWs at high risk from SARS-CoV-2 infection. In this paper, we propose a mechanism for rapid initiation, recruitment and analysis of a large RCT when many of the usual parameters for calculating sample size and efficacy are unknown.

The key design features were the lack of a fixed sample size and no fixed duration of intervention. Given the unknown factors and the need to generate meaningful outcomes as quickly as possible, a frequentist approach to the statistical analysis would be impractical. Frequency statistics test the probability of observing an outcome over the duration of the trial given the true underlying state. In a trial comparing two interventions, one may be considered superior because there is a low probability that the difference would have been observed when the interventions were in fact the same. A sampling distribution of a fixed size is taken and is derived from a known primary outcome variable with its statistical characteristics, and a minimal clinically important difference that might be expected from a new intervention. Statistical analysis usually only occurs at the end of the trial. The resulting *p*-values and confidence intervals (CI) are dependent on sample size. The CIs represent the 95% confidence range for the point estimate of the probability of the outcome occurring while the *p* value represents the chance of observing that outcome when in fact the interventions are the same.

Bayesian analysis asks how likely it is that one intervention is superior to another given the data that has been accumulated during the trial, allowing new data to inform the results of an experiment. There are three underlying statistical concepts. Firstly, conditional probability—the probability of an event (A) given that another (B) has already occurred. Secondly, Bayes theorem, built on the foundation of conditional probability, describes the probability of an event based on prior conditions that might be related to the event. Finally, Bayesian inference, where Bayes’ theorem is used to update the probability of a hypothesis as more evidence (outcomes) become available. Bayesian inference uses a prior probability and a likelihood function derived from a statistical model to derive a posterior probability distribution. In the case of our proposed trial, as cases of SARS-CoV-2 in healthcare workers accumulated we would derive the probability that HCQ reduced SARS-CoV-2 infection in healthcare workers taking HCQ prophylaxis compared to those on no intervention. In summary, the Bayesian approach allows a statistical model to be established in the absence of data generally required for a frequentist model, allows regular adaptive analyses for efficacy and futility and may reduce both the time required to generate a result and the sample sizes required in clinical trials [31].

Other key design features were systems for streamlined baseline data collection and efficient informed consent to ensure the number of participants had minimal impact on overall trial costs. Furthermore, linkage to existing registries where possible, to obtain outcome data, would facilitate the rapid data collection required for the frequent (fortnightly) adaptive analyses required. This approach balanced logistical considerations with the goal of appropriate early stopping from accumulating data if effectiveness or futility was demonstrated, ensuring a conclusion could be obtained as soon as there was sufficient data, rather than when the fixed a priori sample size was reached. Allowing for potential early detection of futility or efficacy of an intervention can be considered a benefit for trials relating to human health, but this could be particularly relevant for the proposed trial given the context in which it was designed (during a rapidly evolving pandemic) and what it sought to determine.

In the event, the international Trial Steering Committee (TSC) made the decision to stop the study before the DSMC had the opportunity to formally meet, due to the marked reduction in COVID-19 cases in NZ and concerns regarding the lack of efficacy and risks with HCQ treatment in COVID-19 from clinical trials in hospitalised patients [2, 23]. Furthermore, in light of the intense media interest in HCQ at the time, survey evidence was collected by the study team which showed that HCWs would not support the planned study, with only 31% of responders in NZ, and less than 13% in IE, indicating willingness to take part in a HCQ study. We have shown, nonetheless, the potential advantages of using a Bayesian statistical approach in early trials investigating possible prophylactic intervention for rapidly evolving pandemics, and the additional benefits that can be derived from proposed trial structure overall.

With only minor changes, the protocol, statistical analysis plan and database developed can be utilised for any potential intervention in the current, or the next, pandemic. One such example could be Baloxavir, a polymerase acidic protein endonuclease inhibitor shown to have efficacy in post-exposure prophylaxis in influenza [32]. With a long half-life that supports infrequent dosing, this medication could replace HCQ in the trial protocol and the same approach could be followed in an influenza pandemic [33]. Additionally, with an effective pre-established protocol, statistical analysis plan, data sharing agreements and CDMAs already in place within an international network of like-minded collaborators, the start-up time from the point of the WHO declaring a public health emergency of international concern to implementation would be greatly reduced and limited only by logistics of drug supply.

### Trial status

The international TSC made the decision to terminate the study before recruitment began.

## Data Availability

Data sharing is not applicable to this article as no datasets were generated or analysed during the study, though the plan was for all datasets generated and/or analysed during the study to be made available from the corresponding author on reasonable request.

## Abbreviations

AE: Adverse event
CDMA: Clinical Data Management Application
CI: Confidence interval
COVID-19: Coronavirus disease 2019 due to SARS-CoV-2 infection
DOT: Directly observed therapy
DSMC: Data and Safety Monitoring Committee
GP: General Practitioner
HCQ: Hydroxychloroquine
HCW: Healthcare worker
HR: Hazard ratio
ICU: Intensive care unit
IE: Ireland
NAAT: Nucleic Acid Amplification Test
NZ: New Zealand
SARS-CoV-2: Severe acute respiratory syndrome coronavirus 2
TSC: Trial Steering Committee
