# Correction: Prophylaxis in healthcare workers during a pandemic: a model for a multi-centre international randomised controlled trial using Bayesian analyses

**DOI:** 10.1186/s13063-022-06499-z

**Published:** 2022-07-05

**Authors:** Pepa Bruce, Kate Ainscough, Lee Hatter, Irene Braithwaite, Lindsay R. Berry, Mark Fitzgerald, Thomas Hills, Kathy Brickell, David Cosgrave, Alex Semprini, Susan Morpeth, Scott Berry, Peter Doran, Paul Young, Richard Beasley, Alistair Nichol

**Affiliations:** 1 Medical Research Institute of New Zealand, Private Bag 7902, Newtown, Wellington, 6242 New Zealand; 2 University College Dublin - Clinical Research Centre at St. Vincent’s University Hospital, Dublin, Ireland; 3 Berry Consultants LLC, Austin, TX USA; 4 Auckland District Health Board, Auckland, New Zealand; 5 National University of Ireland, Galway, Ireland; 6 University Hospital Galway, Galway, Ireland; 7 Counties Manukau District Health Board, Auckland, New Zealand; 8 Monash University - Australian and New Zealand Intensive Care Research Centre, Melbourne, Australia; 9 Department of Intensive Care, Alfred Hospital, Melbourne, Australia

**Correction: Trials 23, 534 (2022)**

**https://doi.org/10.1186/s13063-022-06402-w**

Following the publication of the original article [1], we were notified that the word “Metadata” was mistakenly included in the first author’s name. This has now been removed.

The original article has been corrected.
